# Unintentional fall mortality by place, sex, and age group among older Chinese adults, 2010–21

**DOI:** 10.7189/jogh.14.04170

**Published:** 2024-09-27

**Authors:** Hao Huang, Jingtao Zhou, Min Zhao, Weiqiang Li, David C Schwebel, Zhenzhen Rao, Peishan Ning, Peixia Cheng, Yanhong Fu, Li Li, Guoqing Hu

**Affiliations:** 1 Department of Epidemiology and Health Statistics, Hunan Provincial Key Laboratory of Clinical Epidemiology, Xiangya School of Public Health, Central South University, Changsha City, Hunan Province, China; 2 Department of Psychology, University of Alabama at Birmingham, Birmingham, Alabama, USA; 3 Department of Child, Adolescent and Women's Health, School of Public Health, Capital Medical University, Beijing, China; 4 National Clinical Research Center for Geriatric Disorders, Xiangya Hospital, Central South University, Changsha City, Hunan Province, China

## Abstract

**Background:**

Unintentional falls are known to be a leading cause of injury mortality among older Chinese adults, yet we lack data on the most recent trends in related mortality. To address this, we used the latest nationally representative data from China to examine trends in elderly unintentional fall mortality by place (urban/rural), sex (men/women), and age group (65–69, 70–74, 75–79, 80–84, and ≥85 years) from 2010 to 2021.

**Methods:**

We retrieved mortality data from the Chinese Health Statistical Yearbook (2010–21) and population data from the Chinese Population Census (2010, 2020). Using line graphs, we examined mortality trends over time. We fitted a joinpoint regression model to detect periods experiencing significant changes and calculated the average and specific annual percentage change of mortality rates to quantify significant changes in the mortality of the elderly due to unintentional falls.

**Results:**

Between 2010 and 2021, the age-standardised mortality rate from unintentional falls increased from 45.7 to 67.8 per 100 000 population among Chinese adults aged 65 years and older. Subgroup analyses by sex and place showed similar changing patterns to the overall mortality trends. The joinpoint regression identified certain recent periods that saw significant increases in mortality among adults aged 65–69, 70–74, 75–79, and 80–84 years. During the study period, men and individuals living in rural areas generally had higher unintentional fall mortality rates than women and people living in urban areas (mortality rate ratios: 1.09–1.21 for men vs. women and 1.01–1.27 for rural areas vs. urban areas). Notably, the differences between urban and rural areas, and those between men and women, were consistent across the three younger age groups (65–69, 70–74, and 75–79 years) studied, but reduced in the two oldest age groups (80–84 and ≥85 years).

**Conclusions:**

The age-standardised mortality rate from unintentional falls increased between 2010 and 2021 among Chinese adults aged 65 years or older, with wide variations across years. Unintentional fall mortality has recently increased among adults aged 65 to 84 years. Differences between urban and rural areas, as well as between men and women, deserve the attention of injury researchers and policymakers.

Due to global population ageing and inadequate injury prevention over the last few decades, unintentional falls have become a leading cause of injury morbidity and mortality among older adults in most countries worldwide, including China. According to the Global Burden of Disease (GBD) 2019 estimates, unintentional falls caused approximately 93 360 deaths and 7 814 351 incident cases among Chinese adults aged 65 years and older in 2019 [1]. To address this public health challenge, the government of China has made efforts to reduce elderly falls and fall-induced morbidity and mortality, such as by including unintentional fall prevention among the elderly as a development objective of the Healthy China 2030 initiative [2], and piloting of elderly fall prevention programmes in three cities [3–5].

To monitor the severity of this public health problem and the effect of the efforts launched to address it, trends in elderly unintentional fall morbidity and mortality must be evaluated regularly. To date, several published studies reported changes in mortality due to unintentional falls among the elderly in China based on data from the national Disease Surveillance Points system (DSPs) [6–9], GBD estimates [10], the Chinese Health Statistical Yearbook [11], and published literature [12]. Three studies reported significant increases in elderly unintentional fall mortality over recent years, including findings for adults aged 60 and older from 2013 to 2020 [7] and from 2009 to 2020 [8], and for adults aged 65 years and older from 2003 to 2018 [11]. Conversely, two studies report relatively stable unintentional fall mortality rates among the elderly from 2006 to 2016 [6] and from 1990 to 2019 [10], while one study even reported significant decreases from 2010 to 2016 [9]. The inconsistent results may be due to the use of differing data sources and statistical models. Most previous studies analysed data from DSPs or GBD. Since 2013, the DSPs data set has been expanded to collect mortality data from 605 representative health facilities in China that were selected through multistage sampling [13]. The GBD, in turn, relies primarily on the DSPs and the death ensemble model to perfom mortality estimates in China [14].

Despite conflicting evidence, the epidemiological details of unintentional fall mortality among the elderly in China, particularly by place (urban vs rural), sex, and age group, have not been thoroughly examined in published studies. Using publicly available data, we examined the overall and subgroup unintentional fall mortality among Chinese adults aged 65 years and older between 2010 and 2021 by place, sex, and age group.

## METHODS

### Data source

We used data from the Chinese Health Statistics Yearbook, the only source that reports sex-, place-, and age-specific mortality rates for unintentional falls in China through 2021. It collects nationally representative mortality databased on annual health statistics reports [15]. We focussed on unintentional fall mortality rates from 2010 to 2021.

Because the Chinese Health Statistical Yearbook does not report the numerator and denominator used to calculate the unintentional fall mortality rate, we obtained population data from the Chinese National Population Censuses conducted in 2010 and 2020 [16] and estimated the year-end population for other years during 2010–21. Because the population census is conducted on 1 November and the date that the officially released population applies to is 3 December for each census year, we used linear interpolation to estimate the year-end (31 December) population size for any intercensal year between 2010 and 2020 based on the following formula: N_i_ = N_2010,c_ × w_2010_ + N_2020,c_ × w_2020_; w_2010_ = (1 − d_i,2010_ / 3653); w_2020_ = (1 − d_i,2020_ / 3653) [17].

N_2010,c_ and N_2020,c_ represent the census populations collected on 1 November in 2010 and 2020; w_2010_ and w_2020_ represent the weight of the population in 2010 and 2020; i represents years 2010, 2011, 2012…, 2019; d_i,2010_ represents the number of days between the end of year i and 1 November 2010, and d_i,2020_ is the number of days between the end of year i and 1 November 2020. Note that there were 3653 days between 1 November 2010 and 1 November 2020.

Additionally, due to a lack of data concerning the number of deaths and migration (both immigration and emigration) for subgroup populations by gender, place, and age group, the government used the average annual growth rate of the population from 2010 to 2020 (r) to estimate the year-end population for 2020 and 2021 [18]. The calculation formulas were as follows: r = 1 / 10 × ln (N_2020,c_ / N_2010,c_); N_i_ = N_2010_ × e^r × (yi + m / di)^.

Here, i represents years 2020 and 2021; y_i_ represents the year difference between year i and 2010; m denotes the number of days between the census date (1 November) and the year-end date (31 December) (m = 60); and d_i_ signifies the number of days in year i (d_2020_ = 366, d_2021_ = 365).

Because the Chinese Health Statistics Yearbook only provides annual mortality rates by sex (men vs women), place (urban vs rural), and age group (ages were clustered in five-year groupings, except for <1, 1–4, and ≥85 years), we used the population of 2020 as a reference and calculated the age-standardised unintentional fall mortality for old Chinese adults aged 65 years and older.

### Statistical analysis

We used the age structure of the Chinese population in 2020 to calculate the age-standardised unintentional fall mortality among Chinese adults aged 65 years and older. To do this, we plotted line graphs to show trends in overall and subgroup unintentional fall mortality from 2010 to 2021, and we analysed by place (urban vs rural area), sex (men vs women), and age group (65–69, 70–74, 75–79, 80–84, and ≥85 years).

Using the Joinpoint Regression Program (version 4.9.1.0), we fitted joinpoint regression models to identify periods that experienced significant changes in unintentional fall mortality from 2010 to 2021. We used the age-specific and age-standardised mortality of unintentional falls among the elderly in China from 2010 to 2021 as the dependent variable, with the year as the independent variable, assuming the variance of the dependent variable is stable [19].

The joinpoint regression model is used to determine the best-fit line through several years of data and detect significant turning points within the data, allowing for the analysis of phased changes [19]. The Joinpoint Regression Program adopts an algorithm to test whether a multi-segmented line is a significantly better fit than a straight or less-segmented line, fitting all joinpoint models and then using the sequential permutation test procedure to choose the best one [20]. Additionally, this method assumes that the change in fall mortality rates is constant within each time segment defined by the joinpoints [19]. However, because different subgroups had varying joinpoints in our data, comparing annual percent changes (APCs) across subgroups simultaneously was challenging. To overcome this limitation, we calculated the average annual percent changes (AAPCs) for each subgroup over the study period.

Thus, we used APC and AAPC calculated by joinpoint regression analysis to quantify the significant changes in overall and subgroup unintentional fall mortality rates, and the mortality rate ratio to measure mortality gaps between men and women and between urban and rural areas. The statistical significance threshold was set at *P* < 0.05

## RESULTS

**Figure 1:**
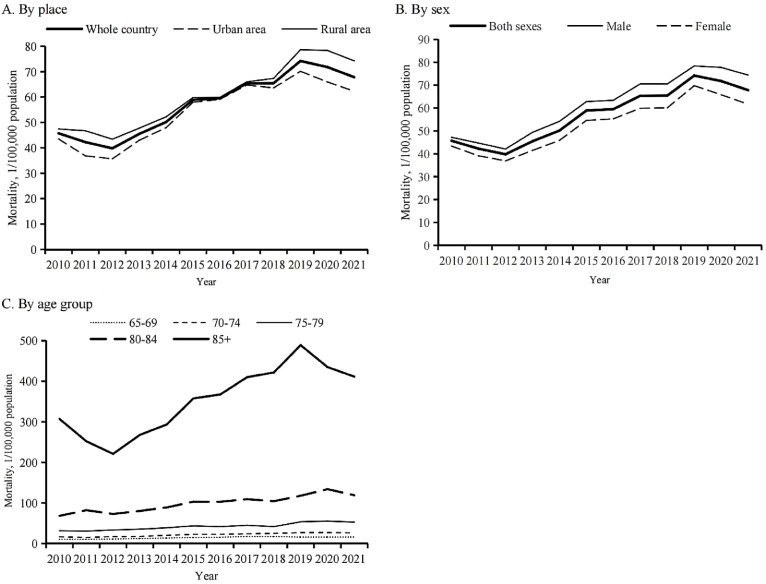
Unintentional fall mortality among Chinese adults aged 65 years and older by place, sex, and age group, 2010–21. **Panel A.** Stratified by place plotted based on age-standardised mortality. **Panel B.** Stratified by sex plotted based on age-standardised mortality. **Panel C**. Stratified by age group plotted based on age-specific mortality.

Between 2010 and 2021, the overall age-standardised unintentional fall mortality in China varied greatly among adults aged 65 years and older. First, it decreased from 45.72 per 100 000 population in 2010 to 39.80 per 100 000 population in 2012, then increased to 74.20 per 100 000 population between 2012 and 2019, before decreasing again to 67.81 per 100 000 population between 2019 and 2021 (**Figure 1**). The AAPC was not statistically significant (AAPC = 4.4%; 95% confidence interval (CI) = −1.4, 10.5) (Table S1 in the **Online Supplementary Document**).

**Figure 2:**
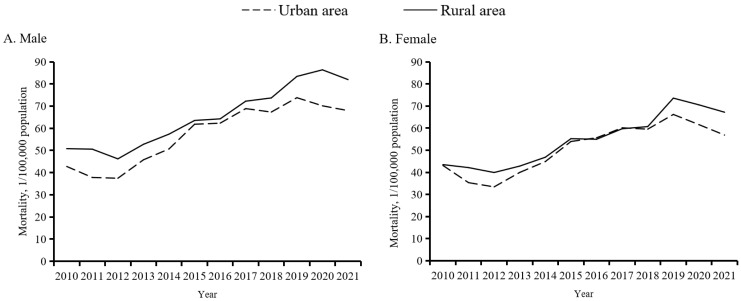
Age-standardised unintentional fall mortality among Chinese adults aged 65 years and older by sex and place, 2010–21. **Panel A.** Analysis for women aged 65 years and older by place. **Panel B.** Analysis for men aged 65 years and older by place.

Surprisingly, the unintentional fall mortality rate ratio between rural and urban areas first decreased from 1.26 to 1.01 in 2010–16, and then gradually rose to 1.19 in 2021 (**Figure 1**, Panel A). The mortality gap between men and women persisted continuously over time and widened as the years passed, with the mortality rate ratio increasing from 1.09 to 1.21 from 2010 to 2021 (**Figure 1**, Panel B). Subgroup analyses in elderly unintentional fall mortality by sex and place showed similar trends for gaps between urban and rural areas and those between men and women, and urban-rural and male-female gaps over time compared to the overall population (**Figure 2**, Panels A–B).

**Figure 3:**
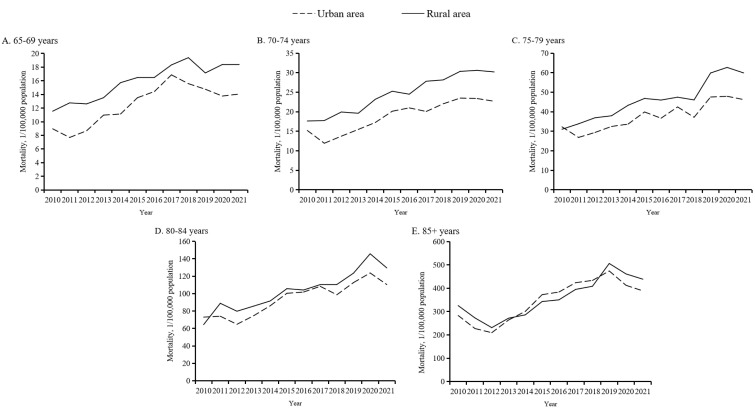
Age-specific unintentional fall mortality among older Chinese adults for five age groups by place, 2010–21. **Panel A.** Analysis for group aged 65–69 years. **Panel B.** Analysis for group aged 70–74 years. **Panel C.** Analysis for group aged 75–79 years. **Panel D**. Analysis for group aged 80–84 years. **Panel E.** Analysis for group aged ≥85 years.

Age-specific unintentional fall mortality increased rapidly as age increased (**Figure 1**, Panel C). The oldest age group, individuals aged 85 years and older, had extremely high mortality rates compared to the four younger age groups. Notably, age-specific analyses only identified significant increases among the 65–69-, 70–74-, 75–79-, and 80–84-year-old subgroups, with respective AAPCs of 4.4% (95% CI = 2.7–6.1), 5.7% (95% CI = 4.4–6.9), 5.6% (95% CI = 4.4–6.8), and 5.7% (95% CI = 4.3–7.1) during 2010 − 21 (Table S1 in the **Online Supplementary Document**). Further analyses by age group and place showed that the trends in overall unintentional fall mortality among the elderly from 2010 to 2021 were mainly driven by mortality changes in the oldest age group (≥85 years) and that the urban-rural gap was large in the three younger age groups (65–69, 70–74, and 75–79 years) and much more modest in the two oldest age groups (80–84 and ≥85 years) (**Figure 3**, Panels A–E).

**Figure 4:**
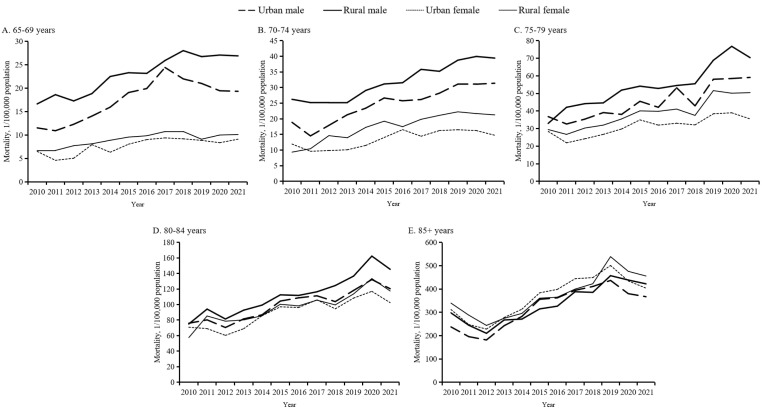
Age-specific unintentional fall mortality among older Chinese adults for five age groups, stratified by place and sex, 2010–21. **Panel A.** Analysis for group aged 65–69 years. **Panel B.** Analysis for group aged 70–74 years. **Panel C.** Analysis for group aged 75–79 years. **Panel D.** Analysis for group aged 80–84 years. **Panel E.** Analysis for group aged ≥85 years.

Detailed age-specific mortality analyses by place and sex showed similar trends, but distinct mortality patterns. All subgroups displayed an increasing tendency (**Figure 4**, Tables S2–3 in the **Online Supplementary Document**). For the three younger groups (65–69, 70–74, and 75–79 years), men in rural areas generally had the highest unintentional fall mortality rates, while women in urban areas had the lowest rates between 2010 and 2021. For the two oldest age groups, gaps across the four subgroups by place and sex narrowed.

## DISCUSSION

### Primary findings

Using nationally representative data, we examined the latest trends in unintentional fall mortality among Chinese adults aged 65 years and older. Four main findings emerged. First, the overall age-standardised unintentional fall mortality varied greatly but generally rose between 2010 and 2021, with the rises driven primarily by mortality changes among the oldest age group (≥85 years). Second, the overall urban-rural gap first narrowed sharply between 2010 and 2016 and then widened gradually between 2016 and 2021. Lastly, the overall gap between men and women increased steadily over time. From 2010 to 2021, rural-residing men generally had the highest unintentional fall mortality rates and urban-dwelling women the lowest mortality rates for the age groups 65–69, 70–74, and 75–79 years, but subgroup mortality gaps by place and sex narrowed for the age groups 80–84 and ≥85 years.

### Interpretation of findings

Despite inconsistencies in the study periods, data sources, and analysis methods between ours and four previous studies [7,8,11,12], we observed similar increases in overall elderly fall mortality from 2010 to 2021. The mortality change in our research was not statistically significant due to fluctuations and the limited number of time points available for joinpoint regression [21]. We found that the observed mortality increase was primarily driven by the mortality rise among adults aged 85 years and older. This may be due to the rapid rise in fall incidence rate among the oldest adults in China. According to GBD 2019 estimates, the fall incidence rate increased from 6.36% in 2010 to 12.26% in 2019 among Chinese adults aged 85 years and older [1]. The observed fall morbidity rate increase has been associated with the higher prevalence of major non-communicable chronic diseases among Chinese adults aged 85 years and older [22,23]. For example, the prevalence of stroke rose by 12% (10 674 to 12 024 per 100 000 population) among Chinese adults aged 85 years and older between 2010 and 2019 [1]. Notably, we observed that the mortality rates due to unintentional falls increased significantly for the four younger age groups (65–69, 70–74, 75–79, and 80–84 years). These increases might be associated with elevated exposure to risk factors such as insufficient lighting, slippery floors, and degenerated physical abilities as a result of non-communicable chronic diseases like osteoporosis, sarcopenia, and multiple chronic comorbidities, as reported in previous research [7,10].

Our findings align with those of a previous report that showed how the unintentional fall mortality gap among the elderly in urban areas and in rural areas narrowed gradually between 2010 and 2016 [9]. However, we also observed a widening urban-rural gap since 2017. Due to a lack of exposure data on key risk factors during the study period (e.g. environmental risk, balance ability, and muscular strength), we cannot confidently attribute this pattern to specific factors. Moreover, our finding of a widening urban-rural gap between 2016 and 2021 is unexpected. It may reflect rising proportions of empty nesters (i.e. older adults living independently without adult children to help them) in rural areas of China [24], since previous research indicates they are at greater risk of falls and related deaths due to lack of family care [25,26]. We also hypothesise that the coronavirus disease 2019 (COVID-19) pandemic seemingly reduced the recent increase in unintentional fall mortality among the elderly. One possible explanation for this result is that strict public health control measures (e.g. travel restrictions) significantly reduced daily activities, consequently lowering the likelihood of activity-related falls.

Our results further follow those of previous reports that older men have a higher fall mortality risk than older women [7]. This gap has been linked to sex-related differences in activities, degradation of physical functions, and the type of fall-induced injuries encountered. Men have higher activity levels and leg muscles that degrade faster, leading to a greater chance of falls [27]. One study has suggested men may sustain more head, face, and chest injuries in falls, while women suffer limb injuries, which might explain a higher fall mortality risk observed in men [28]. Furthermore, the fluctuation of the urban-rural gap during the study period is difficult to interpret. It might be partially associated with changes in data reporting quality. The large unintentional fall mortality gaps we observed between men in rural areas and women in urban areas among the age groups of 65–69, 70–74, and 75–79 years, likely reflect the combined effects of multiple factors discussed above, including daily activities and exposure to fall risks, governmental investment, and degraded physical function. For instance, men in rural areas in their 60s and 70s are more likely to engage in physically intensive occupations and tasks like farm work, while women in urban areas tend to work in less physically intensive activities like domestic housework [29,30]. As people grow older (e.g. ≥80), the impact of many influencing factors diminishes because fewer older adults engage in physical work, and reduced daily activities lead to decreased physical function in all individuals [31–33].

### Policy implications

Our findings have important implications. First, they underscore the significance and urgency of implementing elderly unintentional fall prevention measures nationwide in China. Currently, no national interventions for elderly fall prevention exist, although the national basic public health service programme includes elderly fall prevention as a part of its services [34]. Evidence-based interventions recommended by the World Health Organization, such as balance and gait training with appropriate use of assistive devices, environmental risk assessment and modification, and management of visual deficits [35], should be widely implemented across the country. High-risk populations, including men in rural areas and people aged 85 years and older, should be particularly targeted when the government designs and implements prevention programmes. Additionally, the hard-to-interpret findings from this study, such as the fluctuation of urban-rural mortality differences and inconsistent mortality trends across the five age groups, warrant further research. Future explanatory studies should consider the quality of mortality data, exposure to risk factors, and accessibility and quality of health services, all of which may have been affected by strict public health measures during the COVID-19 pandemic.

### Study limitations

This study has several limitations. First, due to a lack of detailed data (e.g. numerator and denominator of mortality rates) [13], we estimated population size using 2010–20 census-based populations to calculate age-standardised mortality rates. The estimation process might have a slight impact on our results. Second, without data on relevant risk factors and nonfatal unintentional falls, we cannot interpret the causality of observed mortality changes and gaps across subgroups, nor can we generalise results on mortality to nonfatal injury rates. Finally, due to the unavailability of information concerning the quality of mortality data collection and concerning population exposure to specific risk factors for elderly falls such as risky behaviours, low bone density, muscular strength, balance control, and unfriendly living environment, we cannot perform in-depth analyses to interpret the reason for observed elderly unintentional fall mortality changes during the study period.

## CONCLUSIONS

Between 2010 and 2021, unintentional fall mortality among the elderly in China increased by 48% from 45.72 to 67.81 per 100 000 population. The increase was primarily driven by unintentional fall mortality changes among Chinese adults aged 85 years and older. We also observed significant unintentional fall mortality differences across subgroups by place, sex, and age group, with some of these differences changing over time. The government of China should take action to address the growing threat of unintentional falls among the elderly by implementing of proven, cost-effective prevention strategies; supporting research to explore the reasons for and strategies to prevent observed mortality changes and disparities; and tailoring effective interventions from other countries for use in China.

## Additional material


Online Supplementary Document
