# Supplementary material for: Unintentional fall mortality by place, sex, and age group among older Chinese adults, 2010–21
Source: J Glob Health. 2024 Sep 27;14:04170. doi: 10.7189/jogh.14.04170 (PMC11893140; doi:10.7189/jogh.14.04170)
Supplement: Online Supplementary Document [file jogh-14-04170-s001.pdf]

Online Supplementary Documents

Table S1. Annual percent change (APC) and average annual percent change (AAPC) in elderly unintentional fall mortality for both sexes combined between 2010 and 2021 in China

Age group	Place	Block 1		Block 2		Block 3		AAPC (95% CI)
		Time period	APC (95% CI)	Time period	APC (95% CI)	Time period	APC (95% CI)	
≥65 years	Total	2010-2012	-7.0 (-26.5, 17.8)	2012-2015	14.5 (-9.6, 45.0)	2015-2021	3.6 (-0.5, 7.8)	4.4 (-1.4, 10.5)
	Urban	2010-2012	-9.2 (-28.0, 14.6)	2012-2015	19.2 (-5.5, 50.4)	2015-2021	2.0 (-1.9, 6.1)	4.2 (-1.4, 10.2)
	Rural	2010-2012	-3.6 (-16.7, 11.6)	2012-2019	8.2 (5.6, 10.9) *	2019-2021	-1.2 (-14.7, 14.3)	4.2 (1.3, 7.3) *
65-69 years	Total	2010-2017	8.4 (6.4, 10.4) *	2017-2021	-2.1 (-6.3, 2.2)			4.4 (2.7,6.1) *
	Urban	2010-2017	11.1 (7.0, 15.4) *	2017-2021	-4.1 (-12.2, 4.9)			5.4 (1.9,8.9) *
	Rural	2010-2017	6.9 (4.6, 9.2) *	2017-2021	-0.2 (-5.1, 5.0)			4.2 (2.3,6.2) *
70-74 years	Total	2010-2021	5.7 (4.4, 6.9) *					5.7 (4.4, 6.9) *
	Urban	2010-2021	5.9 (4.0, 7.9) *					5.9 (4.0, 7.9) *
	Rural	2010-2021	5.8 (4.8, 6.8) *					5.8 (4.8, 6.8) *
75-79 years	Total	2010-2021	5.6 (4.4, 6.8) *					5.6 (4.4, 6.8) *
	Urban	2010-2021	5.0 (3.3, 6.6) *					5.0 (3.3, 6.6) *
	Rural	2010-2021	6.3 (5.0, 7.5) *					6.3 (5.0, 7.5) *
80-84 years	Total	2010-2021	5.7 (4.3, 7.1) *					5.7 (4.3, 7.1) *
	Urban	2010-2012	-6.3 (-28.7, 23.2)	2012-2015	14.7 (-12.7, 50.8)	2015-2021	2.7 (-2.0, 7.5)	4.1 (-2.5, 11.2)
	Rural	2010-2021	6.1 (4.6, 7.7) *					6.1 (4.6,7.7) *
≥85 years	Total	2010-2012	-11.3 (-26.9, 7.5)	2012-2019	11.2 (7.7, 14.9) *	2019-2021	-9.6 (-25.4, 9.7)	2.8 (-1.0, 6.8)
	Urban	2010-2012	-10.6 (-30.4, 14.9)	2012-2017	16.0 (7.1, 25.6) *	2017-2021	-3.1 (-10.5, 4.9)	3.6 (-1.0, 8.5)
	Rural	2010-2012	-14.3 (-26.6, 0.1)	2012-2019	10.7 (7.9, 13.7) *	2019-2021	-4.8 (-18.5, 11.1)	2.8 (-0.3, 6.0)

*: $P<0.05$.

Note: APC and AAPC were calculated based on Joinpoint regression. Some APCs and AAPCs were not statistically significant due to the limited number of data points although their point estimates were visually notable.

Table S2. Annual percent change (APC) and average annual percent change (AAPC) in elderly unintentional fall mortality for males between 2010 and 2021 in China

Age group	Place	Block 1		Block 2		Block 3		AAPC (95% CI)
		Time period	APC (95% CI)	Time period	APC (95% CI)	Time period	APC (95% CI)	
≥65 years	Total	2010-2012	-5.3 (-22.7, 16.0)	2012-2015	14.4 (-6.6, 40.1)	2015-2021	3.8 (0.3, 7.4) *	4.8 (-0.2, 10.1)
	Urban	2010-2012	-5.9 (-23.1, 15.2)	2012-2015	19.2 (-2.6, 45.9)	2015-2021	2.3 (-1.2, 5.8)	5.0 (0.1, 10.3) *
	Rural	2010-2012	-3.0 (-13.0, 8.1)	2012-2019	8.1 (6.2, 10.1) *	2019-2021	0.1 (-10.3, 11.6)	4.5 (2.3, 6.8) *
65-69 years	Total	2010-2012	2.4 (-11.1, 18.0)	2012-2017	10.4 (5.6, 15.5) *	2017-2021	-2.4 (-6.7, 2.0)	4.1 (1.5, 6.8) *
	Urban	2010-2017	12.2 (9.4, 15.1) *	2017-2021	-4.7 (-10.3, 1.1)			5.7 (3.4, 8.1) *
	Rural	2010-2021	5.1(3.7, 6.4) *					5.1 (3.7, 6.4) *
70-74 years	Total	2010-2021	5.4 (4.3, 6.5) *					5.4 (4.3, 6.5) *
	Urban	2010-2021	6.5 (4.5, 8.5) *					6.5 (4.5, 8.5) *
	Rural	2010-2021	5.0 (4.0, 6.0) *					5.0 (4.0, 6.0) *
75-79 years	Total	2010-2021	6.0 (4.8, 7.1) *					6.0 (4.8, 7.1) *
	Urban	2010-2021	5.5 (3.8, 7.3) *					5.5 (3.8, 7.3) *
	Rural	2010-2021	6.5 (5.0, 8.1) *					6.5 (5.0, 8.1) *
80-84 years	Total	2010-2021	5.8 (4.6, 7.2) *					5.8 (4.6, 7.2) *
	Urban	2010-2021	5.4 (3.9, 7.0) *					5.4 (3.9, 7.0) *
	Rural	2010-2021	6.4 (5.1, 7.8) *					6.4 (5.1, 7.8) *
≥85 years	Total	2010-2012	-11.2 (-29.3, 11.6)	2012-2017	14.7 (6.7, 23.4) *	2017-2021	-0.5 (-7.4, 7.0)	4.0 (-0.3, 8.4)
	Urban	2010-2012	-11.6 (-35.4, 21.0)	2012-2015	27.2 (-7.1, 74.0)	2015-2021	1.1 (-4.1, 6.6)	5.0 (-2.6, 13.3)
	Rural	2010-2012	-12.6 (-27.1, 4.8)	2012-2019	10.7 (7.4, 14.2) *	2019-2021	-3.6 (-19.6, 15.6)	3.4 (-0.2, 7.2)

*: $P<0.05$.

Note: APC and AAPC were calculated based on Joinpoint regression. Some APCs and AAPCs were not statistically significant due to the limited number of data points although their point estimates were visually notable.

Table S3. Annual percent change (APC) and average annual percent change (AAPC) in elderly unintentional fall mortality for females between 2010 and 2021 in China

Age group	Place	Block 1		Block 2		Block 3		AAPC (95% CI)
		Time period	APC (95% CI)	Time period	APC (95% CI)	Time period	APC (95% CI)	
≥65 years	Total	2010-2012	-5.2 (-23.5, 17.6)	2012-2019	9.0 (5.1, 13.0) *	2019-2021	-6.2 (-24.4, 16.3)	3.4 (-0.9, 7.9)
	Urban	2010-2012	-10.5 (-28.2, 11.6)	2012-2016	15.6 (3.5, 29.1) *	2016-2021	0.3 (-4.6, 5.3)	3.4 (-0.9, 8.0)
	Rural	2010-2012	-4.0 (-20.6, 16.1)	2012-2019	8.4 (5.0, 12.0) *	2019-2021	-2.1 (-19.1, 18.4)	4.1 (0.3, 8.1) *
65-69 years	Total	2010-2017	7.6 (3.9, 11.4) *	2017-2021	-2.0 (-9.8, 6.4)			4.0 (0.8, 7.3) *
	Urban	2010-2021	5.4 (2.3, 8.6) *					5.4 (2.3, 8.6) *
	Rural	2010-2017	7.1 (4.7, 9.6) *	2017-2021	-2.4 (-7.4, 3.0)			3.6 (1.5, 5.7) *
70-74 years	Total	2010-2016	10.0 (6.1, 14.0) *	2016-2021	1.5 (-3.2, 6.5)			6.1 (3.5, 8.7) *
	Urban	2010-2012	-11.2 (-30.9, 14.2)	2012-2016	14.8 (1.3, 30.2) *	2016-2021	-0.3 (-5.8, 5.4)	2.8 (-2.2, 7.9)
	Rural	2010-2014	17.1 (8.0, 27.0) *	2014-2021	3.5 (0.0, 7.1) *			8.3 (5.0, 11.6) *
75-79 years	Total	2010-2021	5.1 (3.5, 6.7) *					5.1 (3.5, 6.7) *
	Urban	2010-2021	4.3 (2.4, 6.2) *					4.3 (2.4, 6.2) *
	Rural	2010-2021	6.0 (4.5, 7.5) *					6.0 (4.5, 7.5) *
80-84 years	Total	2010-2021	5.5 (3.9, 7.1) *					5.5 (3.9, 7.1) *
	Urban	2010-2012	-8.2 (-32.0, 23.9)	2012-2015	16.9 (-13.4, 57.8)	2015-2021	2.0 (-3.1, 7.3)	3.8 (-3.4, 11.6)
	Rural	2010-2021	5.8 (3.9, 7.7) *					5.8 (3.9, 7.7) *
≥85 years	Total	2010-2012	-12.4 (-28.3, 7.0)	2012-2019	11.1 (7.4, 14.9) *	2019-2021	-9.5 (-25.9, 10.5)	2.5 (-1.4, 6.6)
	Urban	2010-2012	-12.1 (-32.5, 14.6)	2012-2017	15.2 (6.0, 25.3) *	2017-2021	-2.8 (-10.6, 5.7)	3.1 (-1.7, 8.2)
	Rural	2010-2012	-14.9 (-28.8, 1.9)	2012-2019	10.8 (7.5, 14.2) *	2019-2021	-0.5 (-20.6, 13.6)	2.7 (-0.9, 6.4)

*: $P<0.05$.

Note: APC and AAPC were calculated based on Joinpoint regression. Some APCs and AAPCs were not statistically significant due to the limited number of data points although their point estimates were visually notable.
